# A Reflected-Light-Mode Multiwavelength Interferometer for Measurement of Step Height Standards

**DOI:** 10.3390/s24165082

**Published:** 2024-08-06

**Authors:** Dariusz Litwin, Kamil Radziak, Adam Czyżewski, Jacek Galas, Tadeusz Kryszczyński, Narcyz Błocki, Robert Szumski, Justyna Niedziela

**Affiliations:** 1 Łukasiewicz Research Network-Tele and Radio Research Institute, 11 Ratuszowa St., 03-450 Warsaw, Poland; kamil.radziak@itr.lukasiewicz.gov.pl (K.R.); adam.czyzewski@itr.lukasiewicz.gov.pl (A.C.); jacek.galas@itr.lukasiewicz.gov.pl (J.G.); tadeusz.kryszczynski@itr.lukasiewicz.gov.pl (T.K.);; 2 Time and Length Department, Central Office of Measures, 2 Elektoralna St., 00-139 Warsaw, Poland; robert.szumski@gum.gov.pl (R.S.); justyna.niedziela@gum.gov.pl (J.N.)

**Keywords:** interferometry, variable wavelength interferometry, multiwavelength interferometer, waveplate, refractive index, Wollaston prism, thickness standard, step height standard, metrology

## Abstract

The article is dedicated to measuring the thickness of step height standards using the author’s version of the variable wavelength interferometer (VAWI) in the reflected-light mode, where the interference pattern is created by the combination of two Wollaston prisms. The element of novelty consists in replacing the traditional search for the coincidence of fringes in the object and background with a continuous measurement of their periods and phases relative to the zero-order fringe. The resulting system of sinusoids is then analyzed using two methods: the classical one and the second utilizing the criterion of uniform thickness. The theory is followed by simulation and experimental parts, providing insight to the metrological potential of the VAWI technology.

## 1. Introduction

The interferometric system that utilizes Wollaston prisms integrated with a classical microscope was proposed in the 1980s [1,2,3]. A distinctive feature of these systems has been the quasi-continuously variable wavelength of the illuminating beam. Therefore, the systems are called VAWIs (Variable Wavelength Interferometry). The early systems were not supported by cameras nor were they supported by framegrabbers, and, consequently, the researcher had to manually conduct the measurement procedure, analyzing and counting fringes visually, which was a tedious and slow process. In those days, the application of the system in the industrial environment was not even mentioned in spite of high stability, repeatability, and relatively precise [1,2,3,4,5] measurement results. A collection of variant solutions of the VAWI systems including an early attempt to partly automate it can be found in our cited articles [6,7,8]. In this paper, we concentrate on the classical instrument design with progressively altered wavelength using the Czerny–Turner monochromator. Except our approach, there are many other interferometric systems designed for measuring birefringent objects [9,10] or specific geometrical structures [11,12,13,14], and frequently they use more than one wavelength though their optical characteristics and metrological approach are different. The continuous wavelength attribute of the light source provides simultaneously high accuracy and long measurement range, which constitutes the most important unique feature of the instrument. The interferometer can be configured to measure directional refractive indexes and birefringence [7,8] and, in the case of the waveplates, their retardation. In the reflected-light mode, the interferometer can measure geometrical features of objects like thickness in the case of step height standards. The latter is the leading theme of our paper. In this approach, our intention has been to apply relatively simple algorithms to secure short time of calculations and avoid numerical errors while maintaining high accuracy of measurements. An element of novelty depends on replacing the search for the coincidence and anticoincidence of fringes in the object and background with a continuous measurement of their periods and phases relative to the zero-order fringe of the empty field. The classical method, a development of the traditional variable-wavelength interferometry approach, and the equal-thickness criterion method have been applied to the sinusoidal system thus obtained (the secondary fringes). Our motivation comes from the need to complement the results obtained by the metrological consortium [15] with another type of measuring device, to additionally confirm the results already obtained, and to check the suitability of our interferometer for such reference measurements. The need has been requested by the Central Office of Measures, Warsaw, Poland, a member of the consortium. In addition, the interferometer has some unique advantages. Its multispectral character simultaneously secures the identification of the zero-order fringe and high measurement accuracy. It can be built as a compact device without metrologically significant moving parts.

## 2. Materials and Methods

### 2.1. Optical Architecture

**Figure 1:**
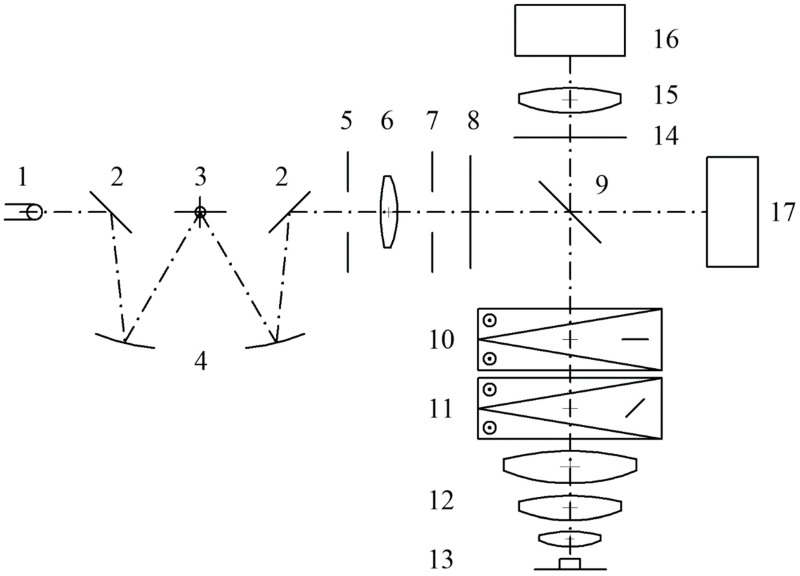
The optical system of the VAWI interferometer. Illumination path: 1—LED, 2—plane mirrors, 3—diffraction grating, 4—spherical mirrors, 5—aperture diaphragm, 6—lens, 7—field diaphragm, 8—polarizer, 9—semitransparent mirror. Imaging path: 10—tube Wollaston prism, 11—objective Wollaston prism, 12—objective, 13—specimen, 14—analyzer, 15—tube lens, 16—CCD camera, 17—auxiliary spectrometer. The elements 2,3,4 constitute the Czerny–Turner monochromator.

**Figure 2:**
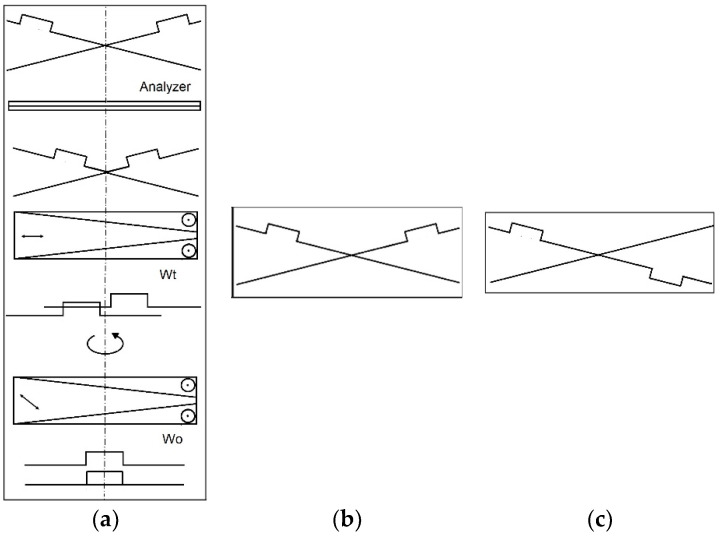
(**a**) Simplified creation of the fringe field by Wollaston prisms Wo and Wt. The polarized plane wave is modified by the reflecting step-like object. (**b**). Final configuration of the interfering waves, where each of them is the reference wave for the other. (**c**). Intuitive, equivalent configuration of the interfering waves. There is one reference wave and one object wave distorted forward and backward.

The optical setup of the interferometric system follows its first prototypes [6]. The current version has been designed for the reflected-light mode, but it can be reconfigured for the transmitted-light one (Figure 1). The system constitutes two arms, i.e., the imaging arm and the illuminating arm. The illuminating arm includes a monochromator (2,3,4), with a white LED as the light source (1), and a polarizer (8). The rest of the optical elements in this arm are analogical to the Köhler principle of the microscope illumination system, i.e., the collector lens (6), aperture (5), and field (7) diaphragms. They are aligned perpendicular to each other. The imaging arm is vertical and consists of a standard microscopic objective (12), the magnification of which is typically equal to 10× or 20×; two Wollaston prisms (10,11); an analyzer (14); and a semitransparent mirror (9). The latter directs the light from the illuminating arm to the imaging arm. In the reflected-light system, the aperture diaphragm is placed closer to the light source, and the field diaphragm is closer to the specimen, whereas in the transmitted-light microscopy, the configuration is reversed. Therefore, the microscope objective has an additional function, i.e., it serves as the condenser for the incoming light. The system is equipped with two Wollaston prisms placed before the objective. The one placed closer to the objective (called the objective prism, 11) splits the original wave into two, which produces the effect of interference between the reference beam and object beams. The other prism (called the tube prism, 10) inclines the waves toward each other at a small angle, generating the fringe field (Figure 2a). It also partly splits the object into its two images.

**Figure 3:**
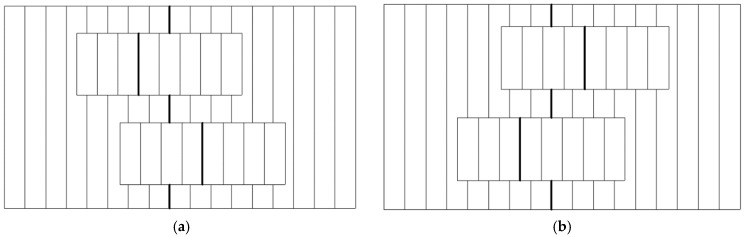
Interference field with a doubled image of the specimen. The bold lines mark the position of the zero-order fringe. (**a**). Wollaston prisms crossed left-handed. (**b**). Wollaston prisms crossed right-handed.

Each wave constitutes the reference wave for the other (Figure 2b), which is equivalent to the interference of one reference wave and one object wave. The latter wave profile includes distortions introduced by the object in two opposite directions: forward and backward (Figure 2c). Schematic fringe fields are presented in Figure 3, where two strips correspond to the doubled image of the single strip-like reflecting object. The fringes in one image are shifted in the opposite direction with respect to the other image. There are two such configurations, i.e., right-handed and left-handed, though in both of them, the objective Wollaston prism and the tube prism are crossed. Such configuration is very stable. The whole unit can work in the industrial environment without any special arrangement. The prism 11 is not necessary in the case of measuring waveplates or should be configured in the subtractive position, i.e., so that the image of the object is not split [1,2,3,4,5]. In such a case, the Wollaston prisms are configured parallel.

### 2.2. Theory

The creation of the fringe field in the VAWI is not intuitive and needs detailed explanation. There are two plane waves orthogonally polarized that reflect from the object (a step height standard) and the substrate. Their spatial positions are consecutively modified by the Wollaston prisms. The objective Wollaston prism splits the waves creating two images of the same object.

The tube prism deflects the waves causing their inclination in relation to each other at a small angle ε. This results in the interference field of straight fringes (Figure 3). Therefore, one-dimensional analysis is sufficient. The split wavefronts can be described as

(1) $$E_{o}=A_{o}\sin(\omega t-\Psi_{o}(x))$$

(2) $$E_{e}=A_{e}\sin(\omega t-\Psi_{e}(x))$$

where A_o_ and A_e_ are the amplitudes of the ordinary and extraordinary waves, respectively; ψ_o_ and ψ_e_ are their phases, respectively; ω = 2π/T, where T is the period of the light wave; and x is the horizontal coordinate. The letter t designates time but in Equations (1) and (2) only.

The intensity in the classical interferometric equation is

(3) $$I\left(x\right)=A_{o}^{2}+A_{e}^{2}+2A_{o}A_{e}\cos(\psi \left(x\right))$$

(4) $$\psi \left(x\right)=\psi_{o}\left(x\right)-\psi_{e}\left(x\right)$$

We assume that the waves have equal amplitude:

(5) $$I(x)=2A^{2}(1+\cos(\psi \left(x\right))$$

**Figure 4:**
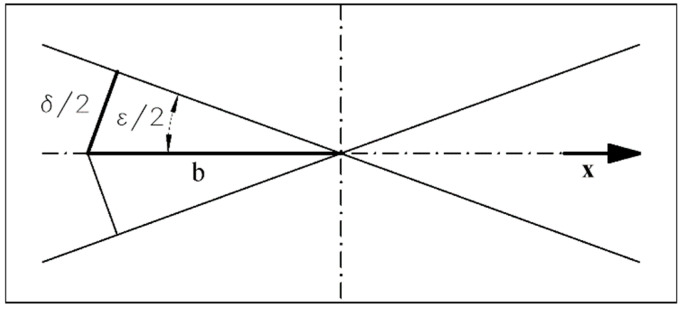
Calculation of the inter-fringe distance b. δ—optical path difference; ε—inclination of the wavefronts.

Naturally, the interference intensity pattern depends on the phase difference, which can be calculated knowing the optical path difference (OPD) δ. From simple geometry (Figure 4):

(6) $$\frac{\delta }{x}=2\sin\left(\frac{\varepsilon }{2}\right)$$

where ε is the angle between interfering component waves, and x is the coordinate characterizing the intensity.

In order to find the inter-fringe distance b, δ = λ:

(7) $$b(\lambda )=\frac{\lambda }{2\sin\left(\frac{\varepsilon (\lambda )}{2}\right)}$$

The intensity can be then given in the following form (taking into account that ε is small enough to neglect the sine function):

(8) $$b(\lambda )=\frac{\lambda }{\epsilon \left(\lambda \right)}$$

and

(9) $$I\left(x,\lambda \right)=2A^{2}\left(1+\cos\left(\frac{2\pi x}{b(\lambda )}+f(x,\lambda )\right)\right)$$

where f(x) characterizes the shift of fringes connected with the object in question.

Substituting b from (8):

(10) $$I\left(x,\lambda \right)=2A^{2}\left(1+\cos\left(\frac{2\pi \varepsilon (\lambda )x}{\lambda }+f(x,\lambda )\right)\right)$$

Alternatively, it is possible to use the following trigonometrical identity:

(11) $$2\cos^{2}\left(\frac{\alpha }{2}\right)=1+\cos(\alpha )$$

receiving finally

(12) $$I\left(x,\lambda \right)=4A^{2}\left(\cos^{2}\left(\frac{\pi \varepsilon (\lambda )x}{\lambda }+\frac{f(x,\lambda )}{2}\right)\right)$$

which is frequently encountered in the literature on interference.

### 2.3. Classical Method

The object under study is positioned in the empty fringe field created by the mentioned pair of two Wollaston prisms. When the wavelength of the illuminating light beam is modified, the inter-fringe distance decreases or increases as well following the corresponding wavelength. The key element in the modified classical approach is to measure the accumulated phase change with respect to the fringes in the empty field or a reference line at each wavelength. In the former case, we can directly measure the optical path difference, whereas in the latter case, we have to take into consideration the position of the zero-order fringe in the empty field. The mentioned phase shift can be calculated by fitting a sine function to the averaged fringe pattern at each wavelength. The measurement process can be conducted in two directions: from the long waves to the short waves or the other way round [1,2,3,8]. Visually, the originator can only observe the coincidence patterns in the fringe field in the object and surrounding media. When the fringes in the object and the empty fringe field created continuous lines, the phase shift can be defined as equal to 2π, which is called the coincidence. The method is possible to be implemented in such a way when the optical path difference introduced by the object ranges from a few to several waves since the observer has to see at least 5–7 coincidences and anticoincidences. For small objects, of thickness close to the wavelength, the observer needs to use a more complex procedure that involves reference lines. Therefore, we abandoned this approach and started to register phase changes not necessarily referring to the coincidences or anticoincidences. The coincidental approach is exhaustively described in the cited Pluta’s publications [1,2,3,4,5] and, with some modifications, recalled in our papers [7,8]. Here, we mention some basic formulas for the convenience of the reader.

**Figure 5:**
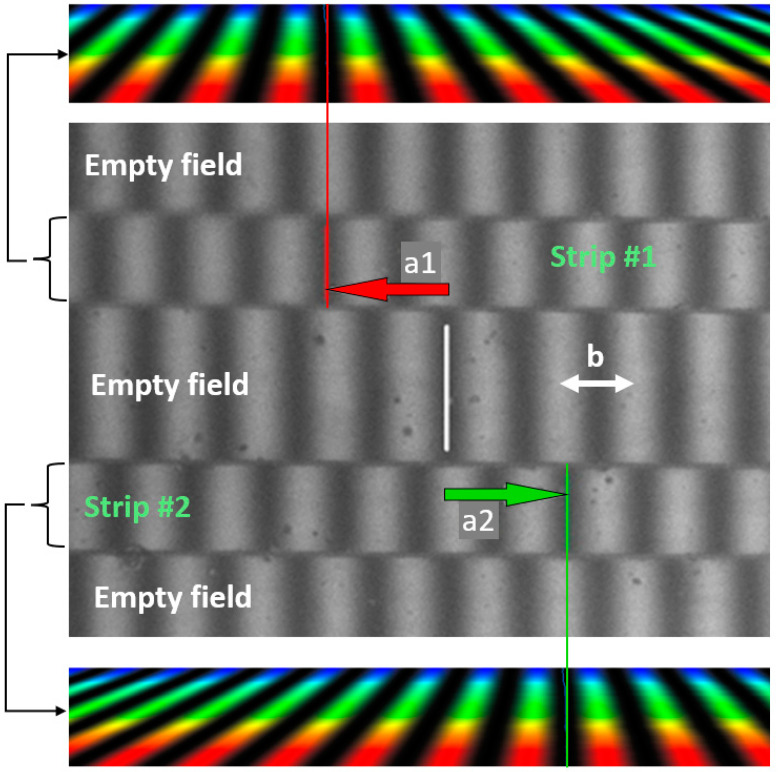
Measurement process in the interference fringe field that utilizes the specific fringe shifts in opposite directions. In color, we illustrate the creation of the secondary fringe pattern that is used to calculate the OPD and, eventually, the thickness of the step height standards.

It can be written that

(13) $$m_{1}\lambda_{1}=2t$$

where m_1_ is the initial interference order characterizing the thickness t of the step height standard at a certain arbitrary wavelength λ_1_. When the wavelength is continuously increased or decreased, the interference order follows this change:

(14) $$\left(m_{1}+q_{s}\right)\lambda_{s}=m_{s}\lambda_{s}=2t$$

where q_s_ is the increment or decrement of the current interference order calculated from the initial phase of the fringe pattern measured in relation to the position of the zero-order fringe in the empty fringe field or a reference line. In order to find values q_s_, our methodology consists of creating a secondary fringe pattern where each line refers to a different wavelength of the primary fringe pattern. As a result, each line is a fitted sinusoid of different initial phase “c” and period “b”. Then, q_s_ = c/2π. These sinusoids are presented in color in Figure 5. For clarity, their colors correspond to their wavelengths.

Solving the system of Equations (13) and (14), we can find the initial interference order:

(15) $$m_{1}=q_{s}\frac{\lambda_{s}}{\lambda_{1}-\lambda_{s}}$$

Alternatively, using the same line of reasoning, it is possible to transform the final equation to the form involving only fringe distances, though the birefringent characteristic of quartz the Wollaston prism is made of must be known anyway.

Substituting (8) and (16) [1]:

(16) $$\varepsilon (\lambda )=4\left[n_{e}\left(\lambda \right)-n_{o}\left(\lambda \right)\right]\tan(\alpha )$$

where α is the apex angle of the tube Wollaston prism. Then, Expression (15) will take the form

(17) $$m_{1}=q_{s}\frac{b_{s}}{{B_{1s}b}_{1}-b_{s}}$$

where

(18) $$B_{1s}=\frac{B_{1}}{B_{s}}$$

and

(19) $$B_{1s}=\frac{\left[n_{e}\left(\lambda_{1}\right)-n_{o}\left(\lambda_{1}\right)\right]}{\left[n_{e}\left(\lambda_{s}\right)-n_{o}\left(\lambda_{s}\right)\right]}=\frac{B_{1}}{B_{s}}$$

Choosing either method, we can calculate m_1_ and immediately the thickness t as

(20) $$t=\frac{m_{1}\lambda_{1}}{2}$$

or

(21) $$t=\left(m_{1}+q_{s}\right)\lambda_{s}/2$$

The thickness calculation according to Formulas (20) or (21) is called the classical method.

However, during the measurement session, we can only measure the inter-fringe distance, and, therefore, we have to find the wavelengths using the calibration plot, i.e., the function b = f(λ), which should be experimentally performed, and will be demonstrated below.

### 2.4. Equal Thickness Method

At the beginning, we recall that, independently of which method is used to calculate the optical path difference, our methodology consists of creating a secondary fringe pattern where each line refers to a different wavelength of the primary fringe pattern. As a result, each line is a sinusoid of different initial phase “c” and period “b” (Figure 5).

Our line of research assumes applying the simplest possible algorithms that would not involve many complex calculations and simultaneously secure low uncertainty and high repeatability. We chose to base our approach on the assumption that the objects are flat with waviness well below the wavelength in use. This approach is called the equal thickness method, ETM, and can be applied either to step height standards or to flat birefringent objects like waveplates.

The optical path difference for a birefringent object can be defined as

(22) $$\frac{a(\lambda )}{2b(\lambda )}\lambda =B(\lambda )t$$

and for a reflecting step height standard

(23) $$\frac{a(\lambda )}{2b(\lambda )}\lambda =t$$

where a(λ) is the zero-order fringe profile, i.e., a set of distances between the zero-order fringe in the object and the empty interference field for the given wavelengths λ (Figure 5); b(λ) is the inter-fringe distance; t is the object thickness; and B(λ) is the object’s birefringence. The denominators are doubled because the light passes the objects twice in the reflected-light mode interferometer.

Initially, we do not know which profile refers to the zero-order fringe, and we assume that an estimated thickness is not known either. Thus, the general function of the profile of the secondary fringe pattern can be written as

(24) $$y_{1}(\lambda )=a_{1}(\lambda )\mp nb(\lambda )$$

(25) $$y_{2}(\lambda )=a_{2}(\lambda )\mp mb(\lambda )$$

Then, using (22), the general thickness functions for a birefringent object will take the following forms:

(26) $$t_{1}^{\prime }\left(\lambda \right)=\frac{a_{1}(\lambda )}{2b(\lambda )B(\lambda )}\lambda \mp \frac{nb(\lambda )}{2b(\lambda )B(\lambda )}\lambda$$

(27) $$t_{1}^{\prime }\left(\lambda \right)=t_{1}\mp \frac{n}{2B(\lambda )}\lambda$$

(28) $$t_{2}^{\prime }\left(\lambda \right)=\frac{a_{2}(\lambda )}{2b(\lambda )B(\lambda )}\lambda \mp \frac{mb(\lambda )}{2b(\lambda )B(\lambda )}\lambda$$

(29) $$t_{2}^{\prime }\left(\lambda \right)=t_{2}\mp \frac{m}{2B(\lambda )}\lambda$$

and for a reflecting object using (23):

(30) $$t_{1}^{\prime }\left(\lambda \right)=\frac{a_{1}(\lambda )}{2b(\lambda )}\lambda \mp \frac{nb(\lambda )}{2b(\lambda )}\lambda$$

(31) $$t_{1}^{\prime }\left(\lambda \right)=t_{1}\mp \frac{n}{2}\lambda$$

(32) $$t_{2}^{\prime }\left(\lambda \right)=\frac{a_{2}(\lambda )}{2b(\lambda )}\lambda \mp \frac{mb(\lambda )}{2b(\lambda )}\lambda$$

(33) $$t_{2}^{\prime }\left(\lambda \right)=t_{2}\mp \frac{m}{2}\lambda$$

The indexes 1 and 2 refer to the secondary fringe pattern shifted in the opposite directions (i.e., to the left and right, see Figure 4 or Figure 5), and m and n describe the secondary fringe profile number counted from the true zero-order fringe. They can be positive or negative.

The algorithm seeks the functions t′, which have the flattest pattern. This occurs when m and n are equal to zero. As the optical system is never entirely symmetrical, we take the average of the found a_1_(λ) and a_2_(λ) and then calculate either the thickness or the spectral characteristic of the waveplate.

(34) $$OPD\left(\lambda \right)=\frac{a_{1}\left(\lambda \right)+a_{2}\left(\lambda \right)}{4b(\lambda )}$$

(35) $$t=\frac{\left[\sum_{i=1}^{k}\frac{a_{1}\left(\lambda_{i}\right)+a_{2}\left(\lambda_{i}\right)}{4b\left(\lambda_{i}\right)}\lambda_{i}\right]}{k}$$

### 2.5. System Calibration

During the measurement session, we measure the inter-fringe distance and find the wavelength using the calibration plot, i.e., the function b = f(λ). The function is theoretically known, and, therefore, it is only necessary to fit it to the actual optical system magnification, which saves a lot of efforts and allows the user to check the system performance every time the sample is replaced and the magnification is adjusted. In this context, it is only necessary to find a scaling factor F since the relation $b=f\left(\lambda \right)$ between the period and the wavelength is known and is proportional to the wavelength and inversely proportional to the birefringence of the material the Wollaston prism is made of. In our instruments, the prisms have been made of quartz.

Substituting (16) to (8), the theoretical function scaled by F takes the form

(36) $$b(\lambda )=F\frac{\lambda }{4\left[n_{e}\left(\lambda \right)-n_{o}\left(\lambda \right)\right]\tan(\alpha )}$$

where α is the apex angle of the tube Wollaston prism.

The quartz birefringence, i.e., $B\left(\lambda \right)=n_{e}\left(\lambda \right)-n_{o}\left(\lambda \right)$, is widely known, and we have taken it from ZEMAX (OpticStudio, v. 17, optical design software) after the Schott constants of dispersion formula.

The factor F depends on the geometrical features of the interferometer and mainly refers to the magnification of the optical system: $b=F\cdot f\left(\lambda \right).$ Thus, we have to minimize the following expression:

(37) $$X^{2}=\sum_{i=1}^{n}\frac{{\left(y_{i}-F\cdot f(\lambda_{i})\right)}^{2}}{\sigma_{i}}$$

where y_i_ is the measured inter-fringe distance at λ_i_.

Assuming that the measurement error σ_i_ is the same over the whole spectrum:

(38) $$\frac{{\partial X}^{2}}{\partial F}=-2\sum_{i=1}^{n}\left(y_{i}-F\cdot f(\lambda ))\cdot f(\lambda \right)$$

(39) $$-\frac{1}{2}\frac{{\partial X}^{2}}{\partial F}=\sum_{i=1}^{n}\left(y_{i}-F\cdot f(\lambda ))\cdot f(\lambda \right)$$

(40) $$-\frac{1}{2}\frac{{\partial X}^{2}}{\partial F}=\sum_{i=1}^{n}\left[y_{i}\cdot f(\lambda )-F\cdot f^{2}(\lambda )\right]$$

(41) $$0=\sum_{i=1}^{n}\left[y_{i}\cdot f(\lambda_{i})-F\cdot f^{2}(\lambda_{i})\right]$$

(42) $$0=\sum_{i=1}^{n}y_{i}\cdot f(\lambda_{i})-F\sum_{i=1}^{n}f^{2}(\lambda_{i})$$

Finally:

(43) $$F=\frac{\sum_{i=1}^{n}\left[y_{i}\cdot f\left(\lambda_{i}\right)\right]}{\sum_{i=1}^{n}\left[f^{2}\left(\lambda_{i}\right)\right]}$$

In our calibration procedure, we used two stabilized lasers emitting light at 532.0 and 632.8 nm, which seems to be sufficient in this approach, though additional light sources are highly recommended.

### 2.6. Software and Simulation

**Figure 6:**
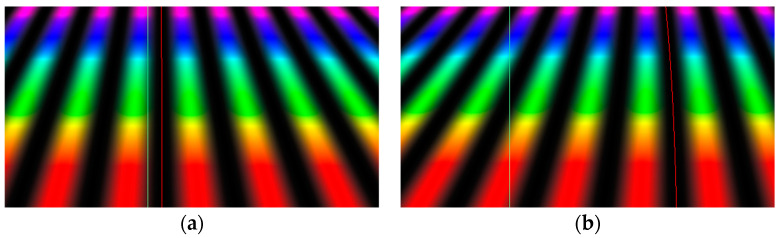
(**a**). Step height standard 70 nm. (**b**). Step height standard 800 nm. A simulated secondary fringe pattern produced for 70 and 800 nm step height standards (390–710 nm spectrum, with the wavelength increment equal to 1 nm) with marked zero-order fringe in empty field and in the object, in green and red, respectively.

Our software includes three independently usable modules. The first module (called VAWI) controls the instrument (the interferometer), its monochromator, and all translation stages. The second module is called VawiViewer, which imports data recorded by the interferometer and calculates the thickness of the step height standards. This piece of software calculates the thickness for all mentioned methods. It also calculates auxiliary and diagnostic data. The third module is the simulation software, called MaterialsLibrary which has the ability to produce artificial data of arbitrary object thickness (Figure 6). These data can be read into the Viewer and can be characterized in the same way as the real data from the interferometer. It is also possible to introduce phase noise imitating in that way the real imperfect data. The noise-free data indicate that either classical or ETM approaches are promising (Table 1). However, the noisy data suggest that the classical method is relatively sensitive to the phase noise, which results in a significant increase in the standard deviation (Table 2). This tendency has been then confirmed in real experiments, where data are not symmetrical like in simulation, which immediately leads to the significant deflection of the mean value (Table 3 and Table 4).

## 3. Results and Discussion

In order to experimentally verify the described methodology, we measured two step height standards with the nominal thicknesses of 70 and 800 nm. We chose these values to test the thickness below and above the average wavelength used in the experiment. The flatness of the surface of both the strips and the substrate were specially prepared to allow different techniques to be applied, including interferometry [15]. The substrate was made of silicon, the measuring plane strips of SiO_2_, and the sample was entirely covered by a chromium layer. The latter eliminates the phase shift between the light reflected from measuring strips and the substrate. The standards were previously manufactured for a different metrological project. In our experiment, we used either the classical or the ETM. Here, we present one of the measuring sessions for each of the standard (Table 3 and Table 4). The measurements were conducted in two Wollaston crossed orientations, i.e., right- and left-handed. What immediately strikes in the first place is that the equal thickness method results are much closer to the averaged values published by the International Consortium [15] of the leading metrological bodies, i.e., 67.53 and 778.39 nm. The results within the Consortium ranged from 65.4 to 68.87 nm, and from 773.7 to 782.8 nm, respectively. The consortium used three different techniques, namely stylus-based, interferometric, and AFM. In the second place, the ETM provides more stable values despite either the monochromator step or the orientation of the Wollaston prisms, and these divergences are significant.

Looking at the dispersion of the results, it can be seen that for all methods, they do not strongly depend on the wavelength increment (monochromator step). Attempting to explain the mismatch between the classical and new approaches, we are inclined to highlight the fact that, in the classical ones, the OPD calculations are based on the accumulated phase (the q measurand in Equations (15), (17) and (21)), which does not guarantee in this implementation that the phase errors would compensate each other entirely during the process of changing wavelengths. Therefore, measurements may produce significantly large error that depends on the system-specific optical and mechanical performance, which, to some extent, can also be repeatable. Conversely, the ETM utilizes the structure of the secondary fringes as a whole and, therefore, provides the best possible match. Analyzing the ultimate accuracy of the system, it must be clearly stressed that it cannot be based on the standard deviation (StD). It characterizes well the dispersion of the results within a single measurement session proving very high repeatedly of the system. However, the interferometric approach utilizes fringe patterns within the specified region of interest. When the sample is repeatedly placed under a microscope, each time it is slightly differently positioned, and, consequently, the thickness variation does not follow the StD measured during a single session when the sample is not moved. In addition, the measured surface height is not perfectly uniform, and, inevitably, the thickness is averaged across the selected region of interest. The same concerns the comparison with the results revealed by the Consortium since neither participant can measure the standards exactly within the same place. In addition, the measuring nature of AFM or stylus instruments is different than that of interferometry. Our experience in this area indicates that the uncertainty can be defined at the level better than ±2 nm, taking into account the above issues. However, considering the high repeatability, we believe that there is significant room for improvement, and the methodology can be further developed.

## 4. Conclusions

We presented a multiwavelength interferometer configured for the reflected-light mode. We showed that the newly implemented equal thickness method (ETM) in this interferometer significantly outperforms the modified classical one (i.e., without searching for coincidences or anticoincidences). The results obtained with the use of this method in this application were closer to the averaged values reported by the European Consortium [15]. However, the classical methodology can be successfully used to find the right profile of the secondary fringe pattern instead of the equal thickness criterion. The application of the interferometer to height step standards should be regarded as exemplary since it can be reconfigured for different objects or materials they are made of, which we theoretically demonstrated. We stress that the nominal thickness need not be known a priori to successfully conduct the measurements. In the most known interferometer systems (e.g., the Twyman–Green or Mach–Zehnder), the reference and object beams propagate along various paths. As a result, vibrations and temperature gradients affect these waves in a different way, which is the reason the fringe pattern is frequently not steady. Thanks to the combination of the Wollaston prisms, the system described in this paper belongs to the common-path interferometer class, where the reference and object waves share the same way. The use of two split images eliminates uncertainty linked to potential asymmetry in the alignment of the system or manufacturing imperfections. In addition, the interferometer includes no moving parts except the rotating grating inside the monochromator, though the latter is metrologically insignificant. The construction of the device is compact and does not require any specific environmental arrangement; thus, it can work even in an industrial location, which is an unquestioned advantage. Therefore, fully automating our system and equipping it with metrologically precise software has been crucial in terms of its practical application and constitutes a significant element of novelty.

## Figures and Tables

**Table 1:** Simulation results for two step height standards of nominal values 70 and 800 nm without noise for two wavelength increments.

Method	70 nm		800 nm	
	Step 1 nm	Step 20 nm	Step 1 nm	Step 20 nm
ETM	70.00000	69.99987	800.00000	799.99846
Classical	70.00000	70.00000	800.00000	800.00000

**Table 2:** Simulation results for two step height standards of nominal values 70 and 800 nm with phase noise (the sinusoid initial phase shift) for conditions imitating the experimental ones.

Equal Thickness Method, ETM		Classical		Session Parameters		
Mean	StD	Mean	StD	Phase Noise [deg]	Δλ [nm]	Spectral Range [nm]
Step height Standard of nominal value 70 nm (67.53 nm EU measurements)						
69.98915	0.04973	69.65957	1.33802	1	1	530–680
69.97646	0.140834	69.75857	0.798263	1	20	530–680
69.92984	0.124557	67.72356	6.580817	4	1	530–680
69.69206	0.25227	70.05661	10.14141	4	20	530–680
Step height Standard of nominal value 800 nm (778.39 nm EU measurements)						
800.00153	0.03596	800.74767	1.56032	1	1	530–680
800.04496	0.06598	799.72384	2.47445	1	20	530–680
799.90762	0.18114	799.61382	7.61079	4	1	530–680
800.01165	0.417622	801.86302	6.60143	4	20	530–680

**Table 3:** Step Height Standard of the nominal value equal to 70 nm.

Equal Thickness Method, ETM		Classical		Session Parameters		
Mean	StD	Mean	StD	Monochro. Step [mm]	Δλ [nm]	Spectral Range [nm]
Right-hand crossed Wollaston prisms						
67.26167	0.00295	69.11500	0.08644	0.025	1	530–680
67.23742	0.00269	68.81043	0.08324	0.050	2	530–680
67.23068	0.00447	68.40731	0.09644	0.100	4	530–680
67.21725	0.00688	68.34139	0.09112	0.500	20	530–680
Left-hand crossed Wollaston prisms						
67.10859	0.00488	65.19099	0.12451	0.025	1	530–680
67.11683	0.00277	64.66593	0.14788	0.050	2	530–680
67.11909	0.00565	63.61196	0.13870	0.100	4	530–680
67.12969	0.01480	58.17638	0.17151	0.500	20	530–680

**Table 4:** Step Height Standard of the nominal value 800 nm.

Equal Thickness Method		Classical		Session Parameters		
Mean	StD.	Mean	StD.	Monochromator Step [mm]	Δλ [nm]	Spectral Range
Right-hand crossed Wollaston prisms						
777.59660	0.01368	785.90332	0.15411	0.025	1	530–680
777.69886	0.00629	785.25151	0.11773	0.050	2	530–680
777.68335	0.00766	785.17949	0.15196	0.100	4	530–680
777.65520	0.01036	788.12045	0.17186	0.250	10	530–680
777.26195	0.01160	794.98908	0.15371	0.500	20	530–680
Left-hand crossed Wollaston prisms						
777.59585	0.00973	797.70729	0.28867	0.025	1	530–680
777.56388	0.00694	799.11664	0.10297	0.050	2	530–680
777.52993	0.00890	801.80489	0.16891	0.100	4	530–680
777.37119	0.00838	798.94157	0.12076	0.250	10	530–680
776.96284	0.01426	784.59328	0.18381	0.500	20	530–680

## Data Availability

Data is contained within the article.
